# Label-Free Electrical Detection Using Carbon Nanotube-Based Biosensors

**DOI:** 10.3390/s90705368

**Published:** 2009-07-08

**Authors:** Kenzo Maehashi, Kazuhiko Matsumoto

**Affiliations:** The Institute of Scientific and Industrial Research, Osaka University, 8-1 Mihogaoka, Ibaraki, Osaka 567-0047, Japan; E-Mail: k-matsumoto@sanken.osaka-u.ac.jp

**Keywords:** label-free biosensors, carbon nanotube, prostate-specific antigen, microfludic chips, field-effect transistors, aptamer, IgE

## Abstract

Label-free detections of biomolecules have attracted great attention in a lot of life science fields such as genomics, clinical diagnosis and practical pharmacy. In this article, we reviewed amperometric and potentiometric biosensors based on carbon nanotubes (CNTs). In amperometric detections, CNT-modified electrodes were used as working electrodes to significantly enhance electroactive surface area. In contrast, the potentiometric biosensors were based on aptamer-modified CNT field-effect transistors (CNTFETs). Since aptamers are artificial oligonucleotides and thus are smaller than the Debye length, proteins can be detected with high sensitivity. In this review, we discussed on the technology, characteristics and developments for commercialization in label-free CNT-based biosensors.

## 1. Introduction

In recent years, there has been a high demand for highly sensitive detections of biomolecules such as DNA, proteins etc., in the many areas of practical pharmacy, genomics, clinical diagnosis for health care, and life sciences [1–5]. In particular, the label-free electrical monitoring of biorecognition events provides a promising platform that is simpler and less expensive and requires less energy than conventional methods. Thus, they are very suitable for minimizing the size of apparatus with biosensors. Rapid testing of different proteins is also required in various applications.

One promising approach to the label-free electrical detection of biomolecules uses carbon nanotubes (CNTs). Since CNTs are formed by rolling graphite sheets, they have quasi-one-dimensional structures. CNTs are one of the most promising materials in terms of fundamental science and technology owing to their unique electrical and mechanical characteristics and nanoscale size. Therefore, they are expected for fabrication of nanoscale electronic devices [6–9].

In this article, we review label-free amperometric and potensiometric biosensors based on CNTs, with special reference to related work carried out in our laboratory. For the amperometric biosensors, CNT-modified electrodes were used as working electrodes. In contract, the potensiometric biosensors were based on CNT field-effect transistors (CNTFETs). In this review, we focused on the technology, characteristics and developments for commercialization in label-free CNT-based biosensors.

## 2. Label-Free Amperometric CNT-Based Biosensors

In general, the three types of electrodes are utilized in electrochemical amperometric sensing: working, reference, and counter electrodes. When enough bias voltage is applied between the working and counter electrodes, electroactive biomolecules in a solution are oxidized or reduced, and then electron transfer reactions occur on the working-electrode surfaces. Therefore, it is important to choose appropriate working electrode materials to achieve electrochemical measurements. CNTs were reported to have the higher ability to promote electron transfer reactions than conventional metal electrodes for electrochemical measurements [10]. Since CNTs have a high aspect ratio, the total surface area of working electrodes becomes larger when CNTs are modified on the surface of electrodes. As a result, highly sensitive detection of biomolecules is expected using CNT-modified electrodes for electrochemical analysis. In this chapter, label-free amperometric biosensors based on CNT electrodes are reviewed. The sensitivity in detection of biomolecules will be improved and the CNT working electrodes can be integrated for multi-biosensors.

### 2.1. Amperometric Biosensors Based on CNT Electrodes

In recent years, amperometric electrochemical biosensors based on CNT-modified electrodes have been fabricated. Fei and co-workers carried out detection of cysteine on Pt/CNT electrodes by cyclic voltammetry [11]. The electrochemical detection of NADH was also demonstrated using scattered-CNT electrodes [12]. Moreover, CNT-modified glassy carbon electrodes were used for the electrochemical measurement of enzymatically generated thiocholine [13].

**Figure 1.**
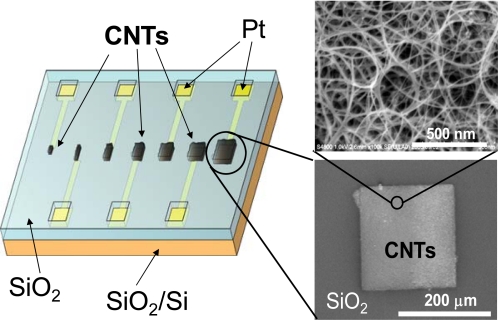
Schematic illustration of the CNT-arrayed electrodes. Inset: a scanning electron microscope (SEM) image of a CNT electrode.

**Figure 2.**
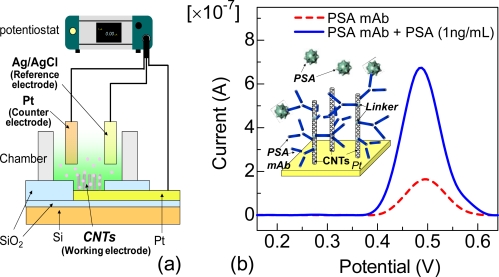
(a) Schematic illustration of experimental setup for the label-free amperometric biosensor. (b) Electrochemical signals obtained from CNT electrodes recorded using DPV. The dotted and solid lines correspond to the electrochemical signals from PSA-mAb and after introduction of 1 ng/mL PSA, respectivity. PSA-mAb was covalently immobilized on the CNTs using linkers.

In this section, we describe amperometric biosensors based on CNT electrodes, which were directly synthesized on Pt surfaces by thermal chemical vapor deposition [14], and were also arrayed on the chip [15], as shown in Figure 1. As a result, the total surface area of working electrodes is much enhanced as compared with that of bare metal electrode. Figure 2(a) shows a schematic illustration of the three electrode system that was applied in the electrochemical measurements. CNT-arrayed electrodes, Pt wire and Ag/AgCl were used as the working, counter and reference electrodes, respectively. The working electrodes were surrounded by a chamber attached on the substrate, as shown in Figure 2(a). The electrochemical characteristics of the devices were investigated using K_3_[Fe(CN)_6_] and electro-active amino acids, indicating that the biosensors based on CNT-arrayed electrodes provided a high sensitivity to detect biomolecules [15].

Label-free amperometric immunosensors based on the CNT electrodes were fabricated to selectivity detect a cancer marker, prostate-specific antigen (PSA). PSA level in serum are dramatically increased in prostate cancer [16,17]. The cut-off limit of PSA between prostate hyperplasia and cancer is 4 ng/mL [18]. The inset in Figure 2(b) shows the schematic structures of the experimental set-up for selective detection of PSA. Monoclonal antibodies against prostate-specific antigen (PSA-mAb) were covalently anchored onto the CNTs using 1-pyrenebutanoic acid succinimidyl ester (Linker). Figure 2(b) shows the electrochemical signals of proteins with the CNT electrodes recorded using differential pulse voltammetry (DPV) [19]. The electrochemical signal was obtained at +0.5 V from only PSA-mAb, as shown in the dotted line in Figure 2(b). After the introduction of 1 ng/mL PSA on the PSA-mAb-modified CNT electrodes, the electrochemical current signal significantly increased, as shown in the solid line in Figure 2(b), indicating that the antigen–antibody complex was formed. The selectivity of the biosensor was also reported using bovine serum albumin as the non-target protein. PSA in the range of 0.25–1 ng/mL can be effectively detected using the CNT electrodes. Since the cut-off limit of PSA between prostate hyperplasia and cancer is 4 ng/mL, the performance of the label-free electrochemical immunosensor seems promising for further clinical applications. Furthermore, the nano-scale features by semiconductor processing made it possible to fabricate arrays with extremely high density and compatibility for further integration.

### 2.2. Microfluidic Chips Based on CNT Electrodes

In this section, microfluidic chips based on the CNT electrodes are reviewed. The micro total analysis system attracted attention worldwide [20–22]. In this system, the units of measurements are integrated using semiconducting fine processes, and all analysis processes are automatically carried out on one chip. It has dual benefits of consuming only a very small amount of reagents for analysis and of markedly reducing analysis time. Significant research and development efforts have been devoted to producing microfluidic chips for the realization of micro total analysis system. In our group, microfluidic chips were fabricated by the combination of amperometric biosensors based on CNT-arrayed electrodes and microchannels with pneumatic micropumps.

**Figure 3.**
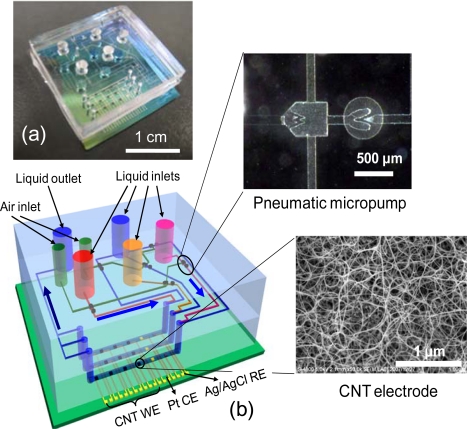
(a) Optical image and (b) schematic illustration of a microfluidic chip based on CNT electrodes. Inset: a SEM image of a CNT electrode and an optical image of a pneumatic micropump.

In microfluidic systems, four kinds of sample solutions can be transported from each liquid inlet into microchannels using six pneumatic micropumps. Figure 3(a),(b) shows the optical and schematic images of the microfluidic chips based on CNT electrodes [23]. There are three channels on the chip, and each channel has four CNT, one platinum and one Ag/AgCl electrodes. One channel contains two inlets and two pneumatic micropumps made of poly(dimethylsiloxane) (PDMS), as shown in inset of Figure 3(b). The pneumatic micropump consisted of three PDMS layers; an air layer, an intermediate membrane and a liquid layer [24]. The air layers of the pneumatic micropumps were connected to the air pressure control. By pulling the air layers of the drive section, the reagents were sucked from inlets to the valve. Subsequently, by pushing them, the reagents pushed out to the electrodes. Repeating that, the reagents were constantly introduced to microchannels. The check valves prevented unexpected reverse flow and diffusion. It can inject 7.8 nL of liquid per cycle.

In the chip, glucose molecules were quantitatively detected by modifying the CNT electrodes with enzyme glucose oxidase [24]. A linear response to glucose concentration within the range from 5 to 20 mg/mL was clearly obtained. Moreover, by controlling the flows of four reagents, two kinds of antibodies; PSA-mAb and human chorionic gonadotropin (hCG) antibody were automatically immobilized onto different CNT electrodes antibodies. Simultaneous detection of two kinds of cancer markers; PSA and hCG were demonstrated using the chip [23]. Microfluidic CNT biochips with pneumatic micropumps are one of the promising platforms for highly sensitive and multi-biosensors of various biomolecules.

## 3. Label-Free Potentiometric CNT-Based Biosensors

**Figure 4.**
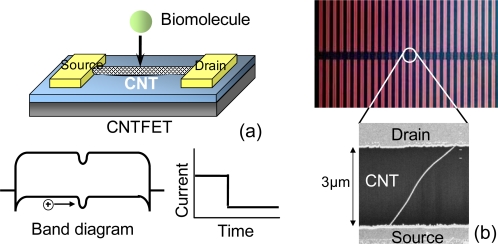
(a) Schematic illustration of detection mechanism for biomolecule using CNTFET. (b) Optical image of arrayed CNTFETs.

CNTs are used for channels in CNTFETs [25], which are notable candidates for highly sensitive label-free biosensors owing to their unique geometries with a high surface-to-volume ratio. Figure 4(a) shows a simple detection mechanism for biomolecules using CNTFETs. When a charged biomolecule adsorbs on the channel of the CNTFET, the CNTFET band structure is modulated. As a result, a reduction in source-drain current is observed. Because the proteins are much larger than the diameter of the CNT channels (1–2 nm), CNTFETs are expected to have a high sensitivity for biomolecule detection.

### 3.1. CNTFETs

The arrayed CNTFETs were fabricated on Si substrates capped with a SiO_2_ layer using position-controlled growth [26,27], as shown in Figure 4(b). CNTs were synthesized by thermal chemical vapor deposition. Source and drain contacts were formed on the patterned chemical catalyst after the CNT growth. The spacing between the source and drain electrodes was approximately 3 μm.

Some groups have investigated the electrical characteristics of CNTFETs which were incubated in ionic solution [28–30]. Reference electrodes were used as the top-gate electrodes. In a solution, the electrical double layer acts as a gate insulator. The results show the good subthreshold and transconductance in the electrolyte-gated CNTFETs, which is due to the large gate capacitance in a solution. Therefore, CNTFETs with an electrolyte in a solution are useful as label-free biosensors.

### 3.2. Biosensors Based on Aptamer-Modified CNTFETs

Biomolecules have been detected using CNTFETs. Star and co-workers reported that by functionalizing CNTs with biotin, streptavidin was detected using CNTFETs [31]. The souce-drain current changed after streptavidin was bonded with CNTs. For DNA sensors, since DNA molecules are negatively charged in a solution, DNA hybridization was detected using CNTFETs [32,33]. For glucose sensors, a conductance in enzyme glucose oxidase modified CNTFETs increased upon addition of glucose [34]. Moreover, Li and co-workers developed immunosensors using CNTFETs for a cancer marker, PSA [35]. PSA-mAb was immobilized with linker and then PSA was detected selectively. Using alternating current measurement, a marked improvement of sensitivity in CNTFET sensors was demonstrated [36].

In this section, we describe label-free detection of immunoglobulin E (IgE) using aptamer-modified CNTFETs. IgE is a subclass of antibodies found only in mammals and exists in human serum at low concentration (∼1 nM), which is only 0.05% of immunoglobulin G concentration. IgE plays a key role in the allergic response, for example, hay fever, atopic dermatitis and allergic asthma, and is especially associated with type 1 hypersensitivity [37,38]. Therefore, it is important for rapid detection of IgE with high sensitivity. The measurement of antigen-antibody reactions is very common in protein detection. However, the typical size of antibodies is around 15 nm [39,40], making them much larger than the Debye length in a buffer solution of typical concentration [41]. As a result, antigen-antibody reactions occur outside the electrical double layer. Thus, it may be difficult to detect proteins with high sensitivity using antibody-modified CNTFETs [42].

**Figure 5.**
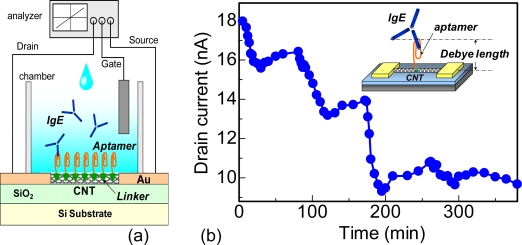
(a) Schematic structure of experimental setup for aptamer-modified CNTFET. (b) Time dependence of drain current in CNTFET after introduction of target IgE at various concentrations into IgE aptamer-modified CNTFET.

In the report, aptamers were used instead of antibodies, as shown in Figure 5(a). Aptamers are artificial oligonucleotides and are produced *in vitro*. Hence, they are less expensive than antibodies but are very stable [43–45]. The greatest advantage of using aptamers is that they are smaller than the Debye length. Thus, protein-aptamer reactions occur inside the electrical double layer. As a result, proteins can be detected with high sensitivity. Moreover, aptamers can also be immobilized at high density on CNT channels. Hence, aptamer-modified CNTFETs have potential for detecting target proteins with high sensitivity. Aptamers were covalently anchored onto the CNTs using 1-pyrenebutanoic acid succinimidyl ester (Linker), as shown in inset of Figure 5(b). An Ag/AgCl reference electrode was used as a gate electrode. Finally, the electrical properties of the CNTFETs were measured in real time using a semiconductor parameter analyzer [42].

**Figure 6.**
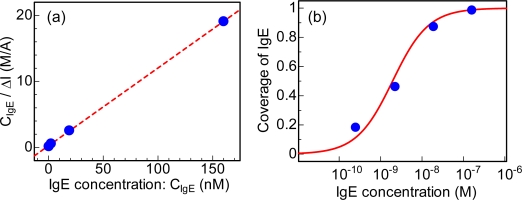
(a) IgE concentration / amount of net drain current as a function of IgE concentration. (b) Coverage of IgE as a function of IgE concentration.

After the introduction of target IgE at various concentrations onto the aptamer-modified CNTFET, the source-drain current sharply decreased, and gradually saturated at lower values, as shown in Figure 5(b). This result indicates that positive charges of IgE molecules were detected by CNT channel conductance modulation in the aptamer-modified CNTFET because IgE-aptamer reactions occur inside the electrical double layer in PBS. Therefore, it is found that IgE in the range of 250 pM–160 nM was effectively detected [42]. From the experimental results, IgE concentration (C_IgE_) / the amount of net drain current (ΔI) was plotted as a function of IgE concentration, as shown in Figure 6(a). This result reveals that the experimental results were fitted well by a linear curve, indicating that the adsorption of IgE onto aptamers on CNT channels follows the Langmuir adsorption isotherm. From the fitting, dissociation constant was estimated to be 1.9 × 10^−9^ M [46]. Compared with the dissociation constants of the reactions between antibodies and antigens for the serum albumin group [47,48], IgE and aptamers have good affinity. The coverage of IgE molecules on the CNT channels was estimated using dissociation constants [46]. The solid line in Figure 6(b) corresponds to the coverage of IgE calculated using the dissociation constant for the reactions between aptamers and IgE. The experimental results in Figure 6(a) were also plotted in Figure 6(b), revealing that the coverage of IgE molecules increased with increasing IgE concentration, namely, the coverage was changed from 18 to 99% at an IgE concentration from 0.25 to 160 nM, respectively.

## 4. Conclusions

In this review, we provide an introduction to label-free amperometric and potentiometric biosensors based on CNTs. In amperometric detections, CNT electrodes, which were directly synthesized on the metal surfaces, were used as working electrodes. The electrochemical measurements indicated that the CNT electrodes have much higher sensitivity to detect biomolecules. Moreover, microfluidic chips based on the CNT electrodes were also reviewed. In contrast, aptamer-modified CNTFETs were fabricated to detect IgE with high sensitivity. It is concluded that label-free CNT-based biosensors are a promising candidate for the development of hand-held electrochemical multiplex biosensors.
